# *Pseudomonas fluorescens*: A Bioaugmentation Strategy for Oil-Contaminated and Nutrient-Poor Soil

**DOI:** 10.3390/ijerph17196959

**Published:** 2020-09-23

**Authors:** Eduardo Jahir Gutiérrez, María del Rosario Abraham, Juan Carlos Baltazar, Guadalupe Vázquez, Eladio Delgadillo, David Tirado

**Affiliations:** 1 Facultad de Ciencias Químico-Biológicas, Universidad Autónoma de Campeche, Av. Agustín Melgar S/N, Buena Vista, Campeche 24039, Mexico; ejgutier@uacam.mx; 2 División de Ciencias de la Vida, Departamento de Ingeniería en Alimentos, Universidad de Guanajuato, Hacienda El Copal Km 9, Carretera Irapuato-Silao; AP 311, Gto. Irapuato 36500, Mexico; mrosarioabdiciva@gmail.com; 3 División de Ingenierías, Departamento de Ingeniería en Minas, Universidad de Guanajuato, Metalurgia y Geología, Ex Had. De San Matias S/N. Col. San Javier, Gto. Guanajuato 36020, Mexico; jc.baltazarvera@ugto.mx; 4 División de Ingenierías, Departmento de Ingeniería Civil y Ambiental, Universidad de Guanajuato, Av. Juárez N 77, Col. Centro, Guanajuato Gto. 36000, Mexico; vazquez.g@ugto.mx (G.V.); e.delgadillo@ugto.mx (E.D.)

**Keywords:** TPH, *Pseudomonas fluorescens*, biodegradation, bioavailability, biosurfactant, surface tension

## Abstract

Bioremediation technology is one of the most profitable and sustainable strategies for remediating soils contaminated with hydrocarbons. This study focuses on assessing the influence of biostimulation and bioaugmentation with *Pseudomonas fluorescens* to contribute to the removal of total petroleum hydrocarbons (TPHs) of a soil. Laboratory studies were carried out (measurements of emitted CO_2_, surface tension, and residual TPH) to select the best bioaugmentation and biostimulation treatment. The sources of C, N, and P were glucose–yeast extract, NH_4_Cl–NaNO_3_, and K_2_HPO_4_–K_3_PO_4_, respectively. The effect of culture conditions on the reduction of TPH and respiratory activity was evaluated through a factorial design, 2^3^, in a solid culture system. After 80 days of incubation, it was observed that treatments of yeast extract–NH_4_Cl–K_2_HPO_4_ (Y4) and glucose–NaNO_3_–K_3_PO_4_ (Y5) presented a higher level of TPH removal (20.91% and 20.00% degradation of TPH, respectively). Biostimulation favors the production of biosurfactants, indirectly measured by the change in surface tension in the soil extracts. The treatments Y4 and Y5 showed a lower change value of the surface tension (23.15 and 23.30 mN·m^−1^ at 25 °C). A positive correlation was determined between the change in surface tension and the removal of TPH; hence there was a contribution of the biosurfactants produced to the removal of hydrocarbons.

## 1. Introduction

In the process of extracting fossil fuels, the industry dedicated to the extraction and refinement of petroleum has generated, per year, hundreds of thousands of tons of soil contaminated with petroleum hydrocarbons [1], which comprise a complex mix of nonaqueous components. Their hydrophobic characteristics make their degradation by the soil microbiota less efficient, resulting in persistent and recalcitrant pollutants in the ecosystem [2]. They are, in rank, number nine on the priority list of substances of the Agency for Toxic Substances and Disease Registry 2017 (ATSDR) [3]. The natural attenuation of hydrocarbon is affected by soil properties like organic matter content, particle size, and the abundance of microbiota [4]. Of the many treatments used for dealing with this type of pollutant, bioremediation technology is one of the most profitable and sustainable strategies for remediating soils contaminated with hydrocarbons [5] and accelerating the natural biodegradation process [6]. Within bioremediation, the two most successful remediation technologies are bioaugmentation and biostimulation, with the former consisting of the addition of a microorganism suspension to the soil. The suspension is taken from the same indigenous microorganisms or from exogenous microorganisms with previously studied degradative capabilities [7,8,9]. Biostimulation involves the addition of nutrients to remove the metabolic limitation of the microbial community and, thus, stimulate its ability to degrade the pollutant [10,11]. Prior research has proven that these two techniques improve the degree to which hydrocarbons can be eliminated from the soil [9,12,13]. Various groups of microorganisms, such as sulfate, nitrate, and iron-reducing archaea or fermentative, syntrophic, and methanogenic archaea, directly or indirectly contribute to the degradation of petroleum hydrocarbons [14]. Several factors need to be taken into account for adequate bioremediation of the polluted soil. First, there are pollutant properties such as polarity, solubility, volatility, and ability to react with other substances. The pollutant concentration defines whether another technology needs to be employed [15]. Second, the following soil properties define the chemical and physical environments: hydrophobicity, pH, moisture content, and organic matter content. The pH affects the solubility, mobility, availability, and ionic forms of a pollutant. Soil moisture is important for oxygen diffusion, but after 60% moisture content, the relative rates of respiration of microbiota decrease [16]. A greater organic matter content can decrease the mobility of organic pollutants [17,18].

Surfactants can be used to increase the bioavailability of petroleum hydrocarbons that are hydrophobic to the aqueous system, reducing the surface tension of the medium and creating two phases with different polarities in the pollutant [19]. The biosurfactants produced by various microorganisms present lower toxicity and, depending on the type of microorganism, a higher level of bioavailability than chemical surfactants [20,21]. Among the most studied are the rhamnolipids, lipopeptides, and phospholipids [19,22]. Biosurfactants have, therefore, been shown to have a wider application for use in the degradation of petroleum hydrocarbons [21,23,24,25]. Various studies can be found in the scientific literature on the segregation and characterization of biosurfactants produced by the *Pseudomonas* genus [26,27]. While some studies have reported the production of biosurfactants in culture medium [28,29], it is important to generate more information on the efficacy of microorganisms to produce biosurfactants in the complex mixture of petroleum hydrocarbons. The objective of this study is to evaluate both the efficiency of total petroleum hydrocarbon (TPH) degradation, applying biostimulation and bioaugmentation strategies with *Pseudomonas fluorescens* ATCC 49642 and the production of biosurfactants in the soil via the simultaneous study of both strategies.

## 2. Materials and Methods

### 2.1. Selection of the Study Area and Soil Sample

For the selection of a sampling site, a place that was contaminated by a hydrocarbon spill and had not received treatment to remediate the soil was located; the site had had a year since a hydrocarbon spill occurred. The soil used in this study was taken from a field belonging to the Escolin petrochemical plant, in the municipality of Poza Rica, Veracruz, México, located in the center of the state at 20°32″ N and 97°27″ E at an altitude of 50 m.a.s.l. This region is considered to have conglomerate soil. The climate is warm, with an annual average temperature of 24.2 °C, annual average precipitation of 1010 mm, and relative humidity of 76% to 80% (INEGI, 2015). Twenty-five soil samples were taken, with a depth range of 0–50 cm and an approximate weight of 4 kg each; all samples were mixed to obtain a composited sample.

### 2.2. Culture Medium and Growth Kinetics of P. Fluorescens

The basal medium for the *P. fluorescens* cell culture was selected in accordance with that implemented by Liu et al. [19], with some modifications, to inoculate *Pseudomonas taiwanensis* and degrade crude oil. The media, contained in milliequivalent water, was prepared with the following nutrients (g·L^−1^): 13.6 glucose 0.01 yeast extract, 1 NH_4_Cl, 5.0 NaNO_3_, 0.2 Fe_2_SO_4_.7H_2_0, 0.2 K_3_PO_4_, 2.0 K_2_HPO_4_, 0.2 MgSO_4_.7H_2_0, 5.0 NaCl, 0.2 KCl, 0.2 CaCl.2H_2_0, 0.006 ZnSO_4_.7H_2_0, 0.1 MnSO_4_.7H_2_0, 0.0006 CoCl_2_.6H_2_0, and 0.0006 CuSO_4_.5H_2_0. The pH of the media was adjusted to 7.0 with NaOH 7 M and then sterilized for 15 min at 121 °C in an autoclave. For the growth kinetics, 100 mL of culture medium was prepared in 250 mL Erlenmeyer flasks and inoculated with the biomass obtained from the washing of two Petri dishes. The sample was incubated at a temperature of 35 °C at 220 rpm for 24 hrs. Three replicates were carried out per sample. Absorbance at 550 nm (VELAB UV/visible spectrophotometer, VE-5100UV) was measured at distinct incubation times in order to determine growth kinetics (hours, and 1, 2, 3, and 4 d). Once the log phase of microbial growth had been studied, the researchers opted to prepare and inoculate the culture medium again, this time taking 10% of the culture medium to inoculate the treatments from the experimental design. In the growth kinetics of *P. fluorescens,* a maximum exponential growth was observed at 48 h of incubation, and it stabilized from 72 h onwards, entering into its stationary phase from the third day onwards. 

### 2.3. Physicochemical Characterization of the Soil

The physicochemical and biological properties of the soil were investigated. Samples were crushed with a mortar and pestle after being air-dried and passed through a 2-mm sieve. Soil moisture was determined by gravimetry. The pH of the soil suspension was measured by potentiometry. The density was made by the pycnometer method. For nitrogen and total phosphorus, they were measured by micro-Kjeldahl and Bray I. Organic matter content was obtained by oxidation with chromic and sulfuric acid. The concentration of TPH present in the soil was calculated by gravimetry. For the concentration of the microbiota present in the soil, the colony-forming units (CFUs) on a plate were counted.

### 2.4. Experimental Design

The maximum and minimum concentrations of the nutrients were selected based on a review of the literature [30,31]. The information on *P. fluorescens* regarding the production of biosurfactants is very limited; for this reason, the selection was based on the nutrients that influence the production of biosurfactants in other microorganisms of the same genus [20,21,30,31,32,33]. To determine the response variables, a 2^3^ factorial design was undertaken and is presented in Table 1. There were two controls: the first containing soil contaminated with TPH and native microbiota, without the addition of nutrients (C1), and the second containing soil contaminated with TPH, *P. fluorescens* inoculum, and native microbiota, without the addition of nutrients (C2). The following continuous variables were in the experimental design: C/N = 100:5; C/P = 100:5; 80 days of incubation; environmental temperature (30–33 °C); 30% humidity, moisture content was monitored every 5 days with a Mengshen dew point temperature and humidity meter; 10% inoculum of *P. fluorescens* at a concentration of 3.2 × 10^6^ CFU with 24 h incubation; each sample contained 3 kg of soil contaminated, the concentration of TPH in soil was 50,000 mg·kg^−1^. Three replicates per treatment were manipulated. The independent variables were the addition of sources of C, N, and P (Table 1). Finally, the response variables measured in this experimental design were residual TPH, surface tension, respiratory activity, and colony-forming units for fungi (CFU_f_) and colony-forming units for bacteria (CFU_b_).

### 2.5. Analytical Methods for the Measurement of the Response Variables

#### 2.5.1. Extraction of Residual TPH from the Soil

The extraction and determination of TPH were carried out according to Mishra et al. [34], with some modifications; 10.0 g of dry soil sample was separately collected from every treatment, mixed with 50 mL dichloromethane (DCM) and 2 g of Na_2_SO_4_. The mixture was shaken in a vortex for a period of 45 s and extracted after 15 min using an ultrasonic processor (JY96-IIN, Ningbo Science Biotechnology CO., LTD, Zhejiang, China), and then centrifuged for 10 min at 6000 rpm. The extraction procedure was repeated three times. The combined extracts were dried using a rotary evaporator at 45 °C, and TPH was then quantified gravimetrically.

#### 2.5.2. Surface Tension

The surface tension was measured using the hanging drop method in a Model 200–00 Standard Goniometer with DROPimage Standard software (ramé-hart instrument co., Succasunna, New Jersey, EEUU) and Young–Laplace software, which is used to describe the shape of the drop under equilibrium conditions [35].

#### 2.5.3. Respiratory Activity (CO_2_)

Gas (CO_2_) analysis was measured using the Gow-Mac Series 580 gas chromatograph, which is equipped with a flame ionization detector (FID) and a capillary column (30 m × 0.32 mm × 40 um). Hydrogen was used as the carrier gas; the oven was at 250 °C, the injector at 250 °C, and the flame ionization detector at 350 °C. A calibration curve was constructed with CO_2_ as a reference standard. All readings were done in duplicate. To carry out the dilutions of the CO_2_ standard, a propylene bag was used, and compressed air was used as a solvent. To inject the samples, a 5 mL SOCOREX syringe for gases was used [36].

#### 2.5.4. Microbial Count

Two groups of microorganisms were evaluated—bacteria and fungi. Nutrient agar (Bioxon, Cuautitlán, Izcalli, Mexico State, Mexico) and rose bengal agar (Difco, Franklin Lakes, New Jersey, USA) were used for the microbial count and for the fungi, respectively. The microorganism count was undertaken using the plate count method for the colony-forming units (CFUs), in which 1 g of soil was weighed and then diluted in 9 mL of sterile saline water (NaCl at 0.9% *w*/*v*). A serial dilution of 1 mL in 9 mL of sterile distilled water was undertaken on the colloidal suspension obtained, with each dilution shaken prior to the next. Once the dilutions (10^−1^ to 10^−8^) had been undertaken, 100 µl was taken from the 10^−2^–10^−4^ solutions to obtain the heterotrophic microorganisms, while 100 µl was taken from the 10^−4^–10^−6^ solutions to obtain the fungi. This was then poured, in sterile conditions, into Petri dishes previously prepared with the medium. Each dilution was inoculated in triplicate. The samples inoculated for the growth of bacteria were incubated at 37 °C for 48 to 72 h, while the Petri dishes inoculated for the growth of fungi were incubated at 28 °C for 7 days in darkness.

### 2.6. Statistical Analysis

The experiments of TPH degradation were carried out in triplicate, results of which represent the mean ± standard deviation. Two-way analysis of variance (ANOVA) and multiple comparisons of means with the LSD and Tukey tests were performed with Minitab Version 18 software. Differences between means at the 5% level (*p* < 0.05) were considered significant. The analysis of the comparison of treatments with the controls was carried out with Dunnett’s tests, HSU, and a response optimizer.

## 3. Results and Discussion

### 3.1. Soil Properties

Characterization of the contaminated soil showed that there was an unfavorable ratio among the principal nutrients, which were carbon, nitrogen, and phosphorous. The phosphorous was not detected (Table 2). The TPH content shows that this was a highly contaminated soil with a slightly alkaline pH, with a moisture content of close to 30% [37], which explains the relationship reported between the moisture content and the degradation of TPH [38]. The indigenous microorganism content in the soil was low (1.04 × 10^4^ CFU); as reported by Kumari et al. [39], the concentration of *P. fluorescens* for the bioaugmentation treatment was 1.2 × 10^7^ colony forming units (CFUs).

The presence of hydrocarbons results in high levels of organic matter content (C), which causes a low concentration of indigenous microorganisms in the soil [40]. A deficiency in both N and P has been reported as one of the limiting factors for the growth of microorganisms capable of degrading pollutants [41] due to the fact that they cannot synthesize compounds such as proteins and nucleic acids, which are essential for microorganism growth, reproduction, and metabolic activity. The low values in the microbial count are due to the fact that not all microorganisms are capable of growing in the presence of hydrocarbons, owing to their structural complexity and their toxicity, so they cannot be degraded by the action of the microbiota. This leads to a decrease in the microbial count compared to noncontaminated soils [40]. Therefore, considering the low microbial concentration identified in the soil featured in this study, researchers opted to apply bioaugmentation using *P. fluorescens* to degrade the hydrocarbons more actively and help the soil recover its properties. Additionally, the biostimulation of the indigenous microorganisms by applying a 2^k^ experimental design to obtain the best response treatment and whether there is synergistic or antagonistic interaction between these two remediation strategies was studied.

### 3.2. Residual TPH

The analysis of variance undertaken on this experimental design revealed that the nutrient sources and their respective interactions presented significant differences in the response variable, which, in this case, is TPH. The values corresponded to a homogeneous distribution and were independent, for which reason they did not depend on a covariate. The nutrients of the soil used were adjusted by applying inorganic fertilizer compounds. According to the test statistics used (LSD and Tukey), the best treatments were Y4, followed by Y5. Reviewing Table 1, where the treatments of the experimental design can be observed, treatment Y4 is composed of the factors yeast extract, NH_4_Cl, and K_2_HPO_4_, and treatment Y5 is composed of glucose, NaNO_3_, and K_3_PO_4_. The Dunnett test results showed that all treatments were significant compared to the control (natural attenuation), with treatments Y4 and Y5 having a greater effect on TPH, with no significant difference found between them. In order to select the best treatment, HSU and response optimizer tests were applied; it was found that Y4 treatment was still the best option (yeast extract–NH_4_Cl–K_2_HPO_4_). It is thus necessary to balance the cost of the glucose, the nutrients, and the quantities added when taking into account the nutrient concentrations.

**Figure 1:**
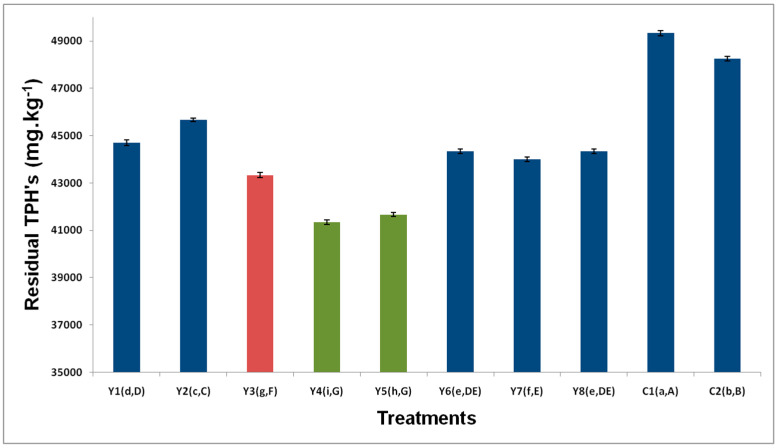
Removal of total petroleum hydrocarbons (TPHs), applying different biostimulation treatments. Standard deviation of the means (Ι). “a,A”: The lower case and uppercase letters in parentheses correspond to the differences between means of the LSD and Tukey tests, respectively.

These ratios of C:N:P were used as they are known to provide the required quantity to convert 100% of petroleum C to cellular biomass [41]. Different behavior was observed in the majority of the biostimulation treatments using different nutrients for the removal of TPH. Figure 1 presents the removal of TPH in the system augmented with *P. fluorescens*, biostimulated with different sources of nutrients, along with their two respective controls (C1—natural attenuation and C2—bioaugmented system without biostimulation). A low degradation rate was observed in C1, which is due to the low concentration of indigenous microorganisms in the soil and this suggests that the application of biostimulation improves the biodegradation of the pollutant in the soil [42]. A low level of TPH removal was observed in Control 2 (C2), a treatment representative of bioaugmentation with *P. fluorescens* in contaminated soil. This is because more than one bacterial species is required to efficiently degrade TPH, as no single microbial species can metabolize the complex mixture of petroleum [8]. A possible option to improve soil bioaugmentation is to add a substrate that allows *P. fluorescens* to form biofilms; in previous studies, it has been observed that the implementation of substrates increases the formation of biofilms of *P. putida*, improving the bioremediation of polluted soil [43,44].

The initial concentration of TPH for all treatments was 50,000 mg·kg^−1^. After an incubation period of 80 days, it was observed that treatments Y4 and Y5 presented a higher level of TPH removal (8667 and 8334 mg·kg^−1^, respectively). Bioremediation depends mainly on soil characteristics and pollutant aging [45]. The presence of high organic matter content and clay may affect the extent of biodegradation due to a priming effect on microbial communities and a decrease in accessibility to microorganisms [46]. The bioavailability of hydrophobic compounds could be reduced by entrapping the compounds into the solid phase of the organic matter [47].

Using a system of biostimulation with inorganic nutrients and bioaugmentation with the *Acinetobacter* strain KF453955, the authors of [48] achieved degradation like that obtained in this investigation, with an initial TPH concentration of 46,000 mg·kg^−1^. Wu et al. showed that biostimulation is more efficient in degrading TPH than bioaugmentation used separately, as inorganic nutrients stimulate microbial growth and, therefore, increase degradative activity [49]. These results agreed with previous studies that concluded that biostimulation is more efficient than bioaugmentation for remediating soils contaminated with hydrocarbons [10,11,50]. In previous studies, the combination of biostimulation and bioaugmentation treatments increased the degradation of TPH in soils [9,13]. Figure 1 shows that the biostimulation process is the most important since the eight treatments received the same amount of *P. fluorescens* inoculum, and, comparing them with control 2 (C2), which corresponds to the bioaugmentation without adding nutrients, the difference in degradation efficiencies in favor of nutrient treatment can be observed. Another observation that can be deduced from these results is that, in reality, the soil studied lacked nutrients, which has prevented the natural attenuation of the pollutant in the soil and resulted in the low degradation rate of the bioaugmentation treatment with *P. fluorescens* [51]. The presence of biosurfactants produced using *P. fluorescens* increases the rates at which pollutants are degraded. Previous studies have quantified biosurfactants in bacteria of the same genus, which improved the availability of the pollutant, as is the case with *Pseudomonas putida* and *aeruginosa* [52,53]. In another study, bioaugmentation of engine-oil-contaminated soil samples with *P. aeruginosa* TPHK-4 resulted in 22% more degradation of hydrocarbons than that occurring in naturally-attenuated samples within 90 days; *P. putida* TPHK-1 also performed better by effecting 18% more degradation of hydrocarbons when compared with natural attenuation [54]. In several studies, the observed increase in TPH removal when soil inoculation was performed was due to different species of *Pseudomonas* (*brassicacearum, mandelii, frederiksbergensis, putida*) with a hydrocarbon-degrading ability [55,56,57,58]. Therefore, the obtained data seems to be consistent with a previous comparative study of Agnello et al. [56] that demonstrated that bioaugmentation was more effective than natural attenuation on the degradation of total petroleum hydrocarbon.

Statistically, the source of nitrogen is the factor that presents the greatest effect on TPH. Nitrogen is considered one of the most important factors due to both its essential role as a cellular constituent and the conservation of metabolic energy [59]. The source of phosphorous has a less significant effect on TPH. The best effect on TPH is achieved with glucose–NH_4_Cl–K_3_PO_4_. It can be deduced that the effects of the interaction among the three sources of biostimulation in relation to TPH are statistically significant. As a source of carbon, glucose presents a higher level of interaction with NaNO_3_ and K_3_PO_4_, while the yeast extract with NH_4_Cl and K_2_HPO_4_ presents a higher level of interaction with TPH. As a source of nitrogen, NaNO_3_ has a higher level of interaction with glucose and K_3_PO_4_, while NH_4_ Cl has a higher level of interaction with yeast extract and K_2_HPO_4_. The addition of nutrients suggests that this is a key strategy for facilitating the degradation process [60].

The three factors, with the respective interactions, influence TPH, with the nitrogen source and carbon source (BC) interactions exercising the greatest effect on TPH. It is observed that the interaction between the three biostimulation sources presents the lowest effect. The source of phosphorous can be eliminated from the treatment and can, thus, reduce the remediation costs.

### 3.3. Surface Tension

Surface tension for the C and N sources were statistically different. Nitrogen had the greatest effect on TPH attenuation. The phosphorous source was less significant in response to the attenuation of TPH. Therefore, the Y4 treatment, which corresponds to yeast extract–NH_4_Cl–K_2_HPO_4_, presented lower surface tension, which translates to the treatment generating the greatest amount of biosurfactant. Yeast extract, as a carbon source, has a greater level of interaction with NH_4_Cl and K_2_HPO_4_. For glucose, no statistical significance was found between nitrogen and phosphorus sources in terms of surface tension. As a source of N, NH_4_Cl has a higher level of interaction with yeast extract and K_2_HPO_4_. Yeast extract–NH_4_Cl–K_2_HPO_4_ had the greatest effect on the study of surface tension.

**Figure 2:**
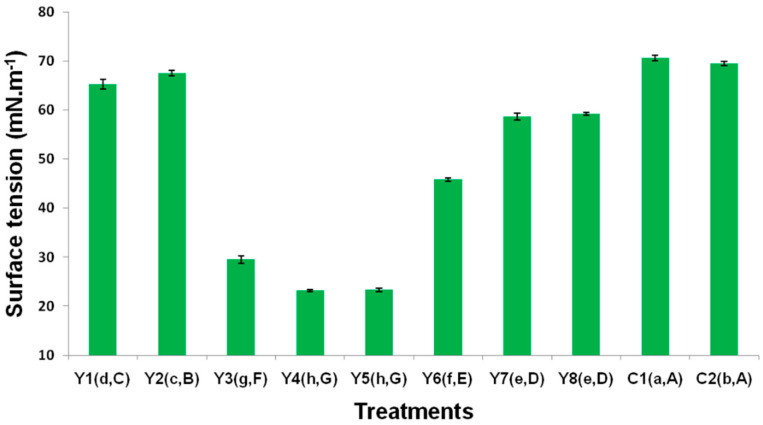
Surface tension values for the different treatments. Standard deviation of the means (Ι). “a,A”: The lower case and upper case letters in parentheses correspond to the differences between means of the LSD and Tukey tests, respectively. Electrolyte solution γ = 71.79 mN/m^−1^ at 25 °C; culture medium γ = 71.93 mN·m^−1^ at 25 °C.

To prove the presence of biosurfactants in the previous systems, an extractant solution was used to measure the changes in the surface tension of the extract in the soils. The initial tension of the systems was considered to be 71.79 mN·m^−1^, while the tension of the culture medium was considered to be 71.93 mN·m^−1^ at a temperature of 25 °C, in accordance with [32]. Figure 2 shows the surface tensions for the different treatments. The treatments that presented lower surface tension were Y4, Y5, and Y3 (23.16, 23.30, and 29.51 mN·m^−1^, respectively). These results are greater than those reported in another study conducted with *P. fluorescens*, which presented a surface tension of 30 to 35 mN·m^−1^ using olive oil as a carbon source and NaNO_3_ as a nitrogen source [39]. The results obtained in this research are less than those reported by Patowary et al. [53], who obtained a surface tension of 25.6 mN·m^−1^ with *P. aeruginosa* SR17 culture, using glucose as a carbon source with an incubation period of 5 days. As can be seen in Figure 2, treatments Y4 and Y5 present a greater level of biosurfactant production as they present values less than the reference values for the electrolyte solution. For this reason, the lower the surface tension, the higher the level of biosurfactant production, with reference values for the electrolyte solution and the culture medium. These surface tension changes confirm the production of biosurfactants by *P. fluorescens*, principally lipoproteins and polymers [61]. The low surface tension has a direct relationship with the removal of TPH, given that previous studies, reporting low surface tension, have found both a higher level of biosurfactant related to increased pollutant removal [38]. Thus, the biosurfactants that may have been produced by *P. fluorescens* could facilitate the solubilization and uptake of insoluble hydrocarbons [62]. These results tend to be similar to those obtained with the response to TPH/TPH, from which it can be deduced that the production of surfactants decreases the surface tension of the pollutant and enables it to degrade at a higher level. Furthermore, the use of biosurfactants to improve the degradation of TPH has a more positive environmental effect than synthetic surfactants [38]. Within the *Pseudomonas* species that degrade hydrocarbons, *P. aeruginosa* and *P. putida* are the best known for hydrocarbon utilization as sources of carbon and energy and for biosurfactant (glycolipid type) production [63]. Biosurfactants improve the desorption of hydrophobic compounds from polluted soil [64]. In the presence of the biosurfactants, there is an increase in the solubilization and desorption of the petroleum hydrophobic hydrocarbons from the soil particles into water [65], facilitating the solubilization and mobilization of the oil compounds [66].

### 3.4. Soil Respiration

**Figure 3:**
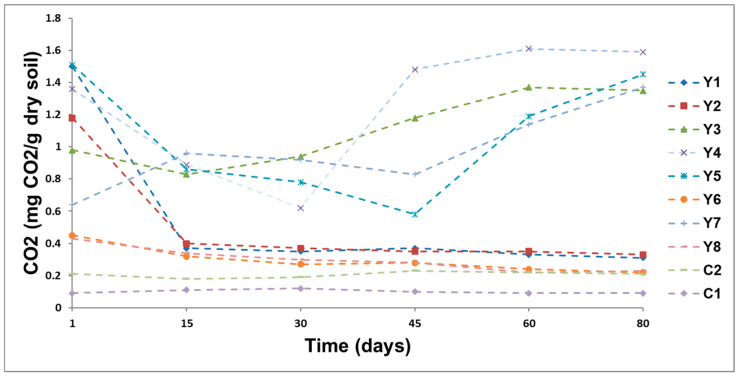
Mean CO_2_ release over 80 days.

The microbial activity was measured using the production of CO_2_ (Figure 3). CO_2_ production in this response was measured over the course of the entire incubation period, with production as a variable across all treatments. The results showed an active microbial population [63]. The treatments presenting the highest levels of CO_2_ production were Y4 > Y5 > Y7 > Y3. The CO_2_ production at different treatments changed in time. The selected nutrients presented an increase in the growth of indigenous microorganisms and *P. fluorescens*. These results suggest that the increase in CO_2_ may be related to the degradation efficiency of TPH.

### 3.5. Population Growth of Bacteria and Fungi

At the end of the experiment, the microorganisms were enumerated. It was observed that the cultivatable bacteria were reactivated across all the treatments (as occurred in other studies reported by [67]), in that microorganism growth was observed under culture conditions of 34–37 °C for 48 hrs in nutrient agar for bacteria and at 28–30 °C for 7 days in rose bengal agar for fungi. This indicates that similar behavior is observed for microbiological latency. The presence of *P. fluorescens* was prominent in comparison to the other colonies by its morphological characteristics (small cocci colonies with a creamy texture and a yellow-beige color). The growth of round transparent bacteria was also observed.

**Figure 4:**
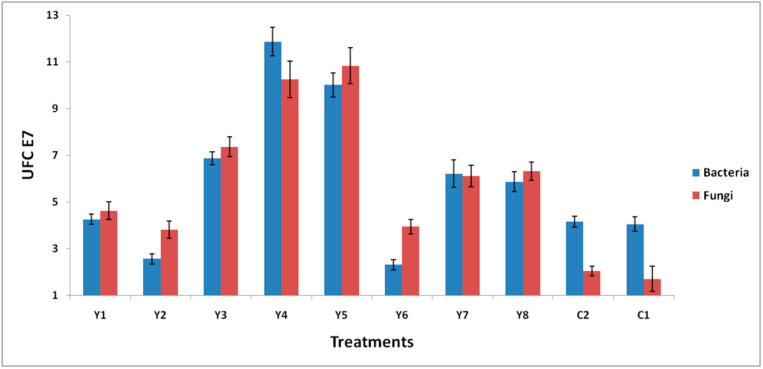
Colony-forming units (CFUs) of fungi and bacteria in the biostimulation system. “Ι” indicates the standard deviation of the means.

Figure 4 shows that the bacteria population is larger for treatments Y4 > Y5 > Y3, which could be due to the addition of *P. fluorescens* as a bioaugmentation system. Various studies have shown that the significant increase in viable bacterial counts is due to the enrichment of the soil because of the nutrient biostimulation [68,69,70,71,72].

However, the fungi population was larger for treatments Y5 > Y4 > Y3, possibly due to the addition of nutrients via the biostimulation strategy, which not only benefitted the bacteria populations but also caused the fungi populations to present higher growth levels. Above all, a synergy effect was observed among the bacterial populations, fungi, and *P. fluorescens.* With no statistical significance found for microbial growth between C1 (natural attenuation) and C2 (addition of *P. fluorescens*), the results of this study reveal that biostimulation is the most effective strategy for the remediation of the soil. From this, it can be concluded that the concentration of nutrients in the attenuated soil is not sufficient to successfully carry out the remediation.

## 4. Conclusions

In this study, we assessed the bioremediation potential of soil contaminated with oil, following different approaches such as natural attenuation, biostimulation, and bioaugmentation. The soil contained high concentrations of hydrocarbons, and natural attenuation was not appreciable; however, soil treatment with CNP nutrients at the recommended levels for biostimulation resulted in a significant increase in natural attenuation, which was even greater for bioaugmentation with *P. fluorescens*. The introduction of a hydrocarbon-degrading bacterial strain, *P. fluorescens*, into hydrocarbon-contaminated soil samples resulted in pronounced bioaugmentation. A combination of both bioaugmentation and biostimulation significantly improved the bioremediation of weathered TPH. 

## Figures and Tables

**Table 1:** Representation of factors and levels, the experimental matrix for the 2^3^ factorial design, the experimentation plan, and the responses measured.

Factors (Independent Variables)						Levels	
Sources of				−1 (g·kg^−1^ of soil)		+1 (g·kg^−1^ of soil)	
X_1_: Source of carbon				13.6 (Glucose)		0.01 (Yeast extract)	
X_2_: Source of nitrogen				5.0 (NaNO_3_)		1.0 (NH_4_Cl)	
X_3_: Source of phosphorous				2.0 (K_2_HPO_4_)		0.2 (K_3_PO_4_)	
**Experiment matrix**				**Experimentation plan**		**Response**	
**Treatment**	**X_1_**	**X_2_**	**X_3_**	**Source of carbon**	**Source of nitrogen**	**Source of phosphorous**	
1	−	−	−	Glucose	NaNO_3_	K_2_HPO_4_	Y_1_
2	+	−	−	Yeast extract	NaNO_3_	K_2_HPO_4_	Y_2_
3	−	+	−	Glucose	NH_4_Cl	K_2_HPO_4_	Y_3_
4	+	+	−	Yeast extract	NH_4_Cl	K_2_HPO_4_	Y_4_
5	−	−	+	Glucose	NaNO_3_	K_3_PO_4_	Y_5_
6	+	−	+	Yeast extract	NaNO_3_	K_3_PO_4_	Y_6_
7	−	+	+	Glucose	NH_4_Cl	K_3_PO_4_	Y_7_
8	+	+	+	Yeast extract	NH_4_Cl	K_3_PO_4_	Y_8_

**Table 2:** The different analytical methods applied to determine the physicochemical parameters of the soil sampled, with each parameter carried out in triplicate.

Parameter	Value	Method
Moisture (%)	32.64 ± 0.46	Gravimetry
pH	7.85 ± 0.01	Potentiometric
Density (kg.cm^3^)	1.09 ± 0.03	Pycnometer
Total nitrogen (%)	0.25 ± 0.00	Micro-Kjeldahl
Total phosphorous (mg·kg^−1^)	n/d	Bray I
Organic matter (%)	11.14 ± 0.26	Oxidation
Texture	Sandy-clay	Hydrometer
TPH (mg·kg^−1^)	50,000 ± 852	Gravimetry
Total bacteria (CFU_b_)	1.04 × 10^4^ ± 3.21 × 10^2^	Plate count
Total fungi (CFU_f_)	1.06 × 10^3^ ± 3.06 × 10^1^	Plate count

The data were mean of triplicates; n/d: not detected, ±: standard deviation. CFU_b_: colony-forming units for bacteria; CFU_f_: colony-forming units for fungi.

## References

[1] Roy A., Dutta A., Pal S., Gupta A., Sarkar J., Chatterjee A., Saha A., Sarkar P., Sar P., Kazy S.K. (2018). Biostimulation and bioaugmentation of native microbial community accelerated bioremediation of oil refinery sludge. Bioresour. Technol..

[2] Maletić S., Dalmacija B.D., Rončević S.D., Agbaba J.R., Perović S.U. (2011). Impact of hydrocarbon type, concentration and weathering on its biodegradability in soil. J. Environ. Sci. Health Part A.

[3] Agency for Toxic Substances and Disease Registry (ATSDR) ATSDR’s Substance Priority List. https://www.atsdr.cdc.gov/spl/.

[4] Abed R.M.M., Al-Kharusi S., Al-Hinai M. (2015). Effect of biostimulation, temperature and salinity on respiration activities and bacterial community composition in an oil polluted desert soil. Int. Biodeterior. Biodegradation.

[5] Cerqueira V.S., Peralba M.C.R., Camargo F.A.O., Bento F.M. (2014). Comparison of bioremediation strategies for soil impacted with petrochemical oily sludge. Int. Biodeterior. Biodegrad..

[6] Coulon F., Brassington K.J., Bazin R., Linnet P.E., Thomas K.A., Mitchell T.R., Lethbridge G., Smith J.W.N., Pollard S.J. (2012). Effect of fertilizer formulation and bioaugmentation on biodegradation and leaching of crude oils and refined products in soils. Environ. Technol..

[7] Andreolli M., Lampis S., Brignoli P., Vallini G. (2015). Bioaugmentation and biostimulation as strategies for the bioremediation of a burned woodland soil contaminated by toxic hydrocarbons: A comparative study. J. Environ. Manag..

[8] Wu M., Chen L., Tian Y., Ding Y., Dick W.A. (2013). Degradation of polycyclic aromatic hydrocarbons by microbial consortia enriched from three soils using two different culture media. Environ. Pollut..

[9] Taccari M., Milanovic V., Comitini F., Casucci C., Ciani M. (2012). Effects of biostimulation and bioaugmentation on diesel removal and bacterial community. Int. Biodeterior. Biodegrad..

[10] Abed R.M.M., Al-Sabahi J., Al-Maqrashi F., Al-Habsi A., Al-Hinai M. (2014). Characterization of hydrocarbon-degrading bacteria isolated from oil-contaminated sediments in the Sultanate of Oman and evaluation of bioaugmentation and biostimulation approaches in microcosm experiments. Int. Biodeterior. Biodegrad..

[11] Sayara T., Borràs E., Caminal G., Sarrà M., Sánchez A. (2011). Bioremediation of PAHs-contaminated soil through composting: Influence of bioaugmentation and biostimulation on contaminant biodegradation. Int. Biodeterior. Biodegrad..

[12] Tahhan R.A., Ammari T.G., Goussous S.J., Al-Shdaifat H.I. (2011). Enhancing the biodegradation of total petroleum hydrocarbons in oily sludge by a modified bioaugmentation strategy. Int. Biodeterior. Biodegrad..

[13] Suja F., Rahim F., Taha M.R., Hambali N., Razali M.R., Khalid A., Hamzah A. (2014). Effects of local microbial bioaugmentation and biostimulation on the bioremediation of total petroleum hydrocarbons (TPH) in crude oil contaminated soil based on laboratory and field observations. Int. Biodeterior. Biodegrad..

[14] Mayumi D., Dolfing J., Sakata S., Maeda H., Miyagawa Y., Ikarashi M., Tamaki H., Takeuchi M., Nakatsu C.H., Kamagata Y. (2013). Carbon dioxide concentration dictates alternative methanogenic pathways in oil reservoirs. Nat. Commun..

[15] Das R., Kazy S.K. (2014). Microbial diversity, community composition and metabolic potential in hydrocarbon contaminated oily sludge: Prospects for in situ bioremediation. Environ. Sci. Pollut. Res..

[16] Linn D.M., Doran J.W. (1984). Effect of Water-Filled Pore Space on Carbon Dioxide and Nitrous Oxide Production in Tilled and Nontilled Soils. Soil Sci. Soc. Am. J..

[17] Tan B., Fowler S.J., Abu Laban N., Dong X., Sensen C.W., Foght J., Gieg L.M. (2015). Comparative analysis of metagenomes from three methanogenic hydrocarbon-degrading enrichment cultures with 41 environmental samples. ISME J..

[18] Ghoreishi G., Alemzadeh A., Mojarrad M., Djavaheri M. (2017). Bioremediation capability and characterization of bacteria isolated from petroleum contaminated soils in Iran. Sustain. Environ. Res..

[19] Liu C., You Y., Zhao R., Sun D., Zhang P., Jiang J., Zhu A., Liu W. (2017). Biosurfactant production from Pseudomonas taiwanensis L1011 and its application in accelerating the chemical and biological decolorization of azo dyes. Ecotoxicol. Environ. Saf..

[20] Aparna A., Srinikethan G., Smitha H. (2012). Production and characterization of biosurfactant produced by a novel Pseudomonas sp. 2B. Colloids Surf. B Biointerfaces.

[21] Al-Wahaibi Y.M., Joshi S., Al-Bahry S., Elshafie A., Al-Bemani A., Shibulal B. (2014). Biosurfactant production by Bacillus subtilis B30 and its application in enhancing oil recovery. Colloids Surf. B Biointerfaces.

[22] Sha R., Meng Q. (2016). Antifungal activity of rhamnolipids against dimorphic fungi. J. Gen. Appl. Microbiol..

[23] Pantsyrnaya T., Blanchard F., Delaunay S., Goergen J.-L., Guedon E., Guseva E., Boudrant J. (2011). Effect of surfactants, dispersion and temperature on solubility and biodegradation of phenanthrene in aqueous media. Chemosphere.

[24] Wyrwas B., Chrzanowski Ł., Ławniczak Ł., Szulc A., Cyplik P., Białas W., Szymański A. (2011). Utilization of Triton X-100 and polyethylene glycols during surfactant-mediated biodegradation of diesel fuel. J. Hazard. Mater..

[25] Szulc A., Ambrożewicz D., Sydow M., Ławniczak Ł., Piotrowska-Cyplik A., Marecik R., Chrzanowski Ł. (2014). The influence of bioaugmentation and biosurfactant addition on bioremediation efficiency of diesel-oil contaminated soil: Feasibility during field studies. J. Environ. Manag..

[26] Santos D.K.F., Rufino R.D., Luna J.M., Santos V.A., Sarubbo L. (2016). Biosurfactants: Multifunctional Biomolecules of the 21st Century. Int. J. Mol. Sci..

[27] Sarubbo L., Rocha R., Luna J., Rufino R., Santos V., Banat I.M. (2015). Some aspects of heavy metals contamination remediation and role of biosurfactants. Chem. Ecol..

[28] Peng F., Wang Y., Sun F., Liu Z., Lai Q., Shao Z. (2008). A novel lipopeptide produced by a Pacific Ocean deep-sea bacterium, Rhodococcussp. TW53. J. Appl. Microbiol..

[29] Prieto L., Michelon M., Burkert J., Kalil S.J., Burkert C. (2008). The production of rhamnolipid by a Pseudomonas aeruginosa strain isolated from a southern coastal zone in Brazil. Chemosphere.

[30] Kitamoto D., Isoda H., Nakahara T. (2002). Functions and potential applications of glycolipid biosurfactants — from energy-saving materials to gene delivery carriers. J. Biosci. Bioeng..

[31] Chayabutra C., Wu J., Ju L.W. (2001). Rhamnolipid production by *Pseudomonas aeruginosa* under denitrificacion: Effects of limiting nutrients and carbón substrates. Biotechnol. Bioeng..

[32] Haftka J., Hammer J., Hermens J.L.M. (2015). Mechanisms of Neutral and Anionic Surfactant Sorption to Solid-Phase Microextraction Fibers. Environ. Sci. Technol..

[33] Marchant R., Banat I.M. (2012). Biosurfactants: A sustainable replacement for chemical surfactants?. Biotechnol. Lett..

[34] Mishra S., Jyot J., Kuhad R., Lal B. (2001). In situ bioremediation potential of an oily sludge-degrading bacterial consortium. Curr. Microbiol..

[35] Berry J.D., Neeson M.J., Dagastine R.R., Chan D., Tabor R., Berry J.D. (2015). Measurement of surface and interfacial tension using pendant drop tensiometry. J. Colloid Interface Sci..

[36] Romero H., Acaro J., Camacho A., Castillo A., Vega C., Dávila K., Gadvay K. (2017). Reliability of a method for determining CO_2_ by gas chromatography. J. Cumbres.

[37] Abouseoud M., Maachi R., Amrane A., Boudergua S., Nabi A. (2008). Evaluation of different carbon and nitrogen sources in production of biosurfactant by *Pseudomonas fluorescens*. Desalination.

[38] Jiang Y., Brassington K.J., Prpich G.P., Paton G.I., Semple K.T., Pollard S.J., Coulon F. (2016). Insights into the biodegradation of weathered hydrocarbons in contaminated soils by bioaugmentation and nutrient stimulation. Chemosphere.

[39] Silva R.D.C.F.S.D., Almeida D.G., Meira H.M., Silva E.J., Farias C.B., Rufino R.D., Luna J.M., Sarubbo L. (2017). Production and characterization of a new biosurfactant from Pseudomonas cepacia grown in low-cost fermentative medium and its application in the oil industry. Biocatal. Agric. Biotechnol..

[40] Kumari S., Wati L., Kant R., Singh U. (2017). Effect of Mixed Bioinocula on Growth and Efficiency of Azotobacter Species. Int. J. Curr. Microbiol. Appl. Sci..

[41] Atlas R.M. (1995). Petroleum biodegradation and oil spill bioremediation. Mar. Pollut. Bull..

[42] Dibble J.T., Bartha R. (1979). Effect of environmental parameters on the biodegradation of oil sludge. Appl. Environ. Microbiol..

[43] Farber R., Dabush-Busheri I., Chaniel G., Rozenfeld S., Bormashenko E., Multanen V., Cahan R. (2019). Biofilm grown on wood waste pretreated with cold low-pressure nitrogen plasma: Utilization for toluene remediation. Int. Biodeterior. Biodegrad..

[44] Farber R., Rosenberg A., Rozenfeld S., Banet G., Cahan R. (2019). Bioremediation of Artificial Diesel-Contaminated Soil Using Bacterial Consortium Immobilized to Plasma-Pretreated Wood Waste. Microorganisms.

[45] Liu P.-W.G., Chang T.C., Chen C.-H., Wang M.-Z., Hsu H.-W. (2013). Effects of soil organic matter and bacterial community shift on bioremediation of diesel-contaminated soil. Int. Biodeterior. Biodegrad..

[46] Hassan I.A., Mohamedelhassan E., Yanful E.K., Weselowski B., Yuan Z.-C. (2019). Isolation and characterization of novel bacterial strains for integrated solar-bioelectrokinetic of soil contaminated with heavy petroleum hydrocarbons. Chemosphere.

[47] Chen Y.-A., Liu P.-W.G., Whang L.-M., Wu Y.-J., Cheng S.-S. (2020). Effect of soil organic matter on petroleum hydrocarbon degradation in diesel/fuel oil-contaminated soil. J. Biosci. Bioeng..

[48] Millioli V.S., Servulo E.L.C., Sobral L.G.S., De Carvalho D.D. (2009). Bioremediation of crude oil bearing soil: Evaluating the effect of rhamnolipid addition to soil toxicity and to crude oil biodegradation efficiency. Glob. Nest J..

[49] Wu M., Dick W.A., Li W., Wang X.C., Yang Q., Wang T., Xu L., Zhang M., Chen L. (2016). Bioaugmentation and biostimulation of hydrocarbon degradation and the microbial community in a petroleum-contaminated soil. Int. Biodeterior. Biodegrad..

[50] Atlas R.M., Hazen T.C. (2011). Oil Biodegradation and Bioremediation: A Tale of the Two Worst Spills in U.S. History. Environ. Sci. Technol..

[51] Kauppi S., Sinkkonen A., Romantschuk M. (2011). Enhancing bioremediation of diesel-fuel-contaminated soil in a boreal climate: Comparison of biostimulation and bioaugmentation. Int. Biodeterior. Biodegrad..

[52] Mariano A., Kataoka A.P.D.A.G., Angelis D.D.F.D., Bonotto D.M. (2007). Laboratory study on the bioremediation of diesel oil contaminated soil from a petrol station. Braz. J. Microbiol..

[53] Patowary R., Patowary K., Kalita M.C., Deka S. (2018). Application of biosurfactant for enhancement of bioremediation process of crude oil contaminated soil. Int. Biodeterior. Biodegrad..

[54] Cha M., Lee N., Kim M., Kim M., Lee S.-J. (2008). Heterologous production of Pseudomonas aeruginosa EMS1 biosurfactant in Pseudomonas putida. Bioresour. Technol..

[55] Ramadass K., Megharaj M., Venkateswarlu K., Naidu R. (2018). Bioavailability of weathered hydrocarbons in engine oil-contaminated soil: Impact of bioaugmentation mediated by Pseudomonas spp. on bioremediation. Sci. Total Environ..

[56] Agnello A., Bagard M., Van Hullebusch E.D., Esposito G., Huguenot D. (2016). Comparative bioremediation of heavy metals and petroleum hydrocarbons co-contaminated soil by natural attenuation, phytoremediation, bioaugmentation and bioaugmentation-assisted phytoremediation. Sci. Total Environ..

[57] Ji Y., Mao G., Wang Y., Bartlam M. (2013). Structural insights into diversity and n-alkane biodegradation mechanisms of alkane hydroxylases. Front. Microbiol..

[58] Liu T., Wang F., Guo L., Li X., Yang X., Lin A.-J. (2012). Biodegradation of n-hexadecane by bacterial strains B1 and B2 isolated from petroleum-contaminated soil. Sci. China Chem..

[59] Zhang X., Xu D., Zhu C., Lundaa T., Scherr K.E. (2012). Isolation and identification of biosurfactant producing and crude oil degrading *Pseudomonas aeruginosa* strains. Chem. Eng. J..

[60] Sarkar D., Ferguson M., Datta R., Birnbaum S. (2005). Bioremediation of petroleum hydrocarbons in contaminated soils: Comparison of biosolids addition, carbon supplementation, and monitored natural attenuation. Environ. Pollut..

[61] Hejazi R.F., Husain T. (2004). Landfarm Performance under Arid Conditions. 2. Evaluation of Parameters. Environ. Sci. Technol..

[62] Almansoory A.F., Abu Hasan H., Abdullah S.R.S., Idris M., Anuar N., Al-Adiwish W.M. (2019). Biosurfactant produced by the hydrocarbon-degrading bacteria: Characterization, activity and applications in removing TPH from contaminated soil. Environ. Technol. Innov..

[63] Shekhar S., Sundaramanickam A., Balasubramanian T. (2014). Biosurfactant Producing Microbes and their Potential Applications: A Review. Crit. Rev. Environ. Sci. Technol..

[64] Pourfadakari S., Moghadam M.A., Jaafarzadeh N., Takdastan A., Neisi A.A., Ghafari S., Jorfi S., Jaafarzadeh N. (2019). Remediation of PAHs contaminated soil using a sequence of soil washing with biosurfactant produced by Pseudomonas aeruginosa strain PF2 and electrokinetic oxidation of desorbed solution, effect of electrode modification with Fe3O4 nanoparticles. J. Hazard. Mater..

[65] Fanaei F., Moussavi G., Shekoohiyan S. (2020). Enhanced treatment of the oil-contaminated soil using biosurfactant-assisted washing operation combined with H2O2-stimulated biotreatment of the effluent. J. Environ. Manag..

[66] Lai C.-C., Huang Y.-C., Wei Y.-H., Chang J.-S. (2009). Biosurfactant-enhanced removal of total petroleum hydrocarbons from contaminated soil. J. Hazard. Mater..

[67] Das N., Chandran P. (2011). Microbial Degradation of Petroleum Hydrocarbon Contaminants: An Overview. Biotechnol. Res. Int..

[68] Diplock E.E., Mardlin D., Killham K., Paton G. (2009). Predicting bioremediation of hydrocarbons: Laboratory to field scale. Environ. Pollut..

[69] Covino S., Čvančarová M., Muzikář M., Svobodová K., D’Annibale A., Petruccioli M., Federici F., Křesinová Z., Cajthaml T. (2010). An efficient PAH-degrading *Lentinus* (Panus) *tigrinus* strain: Effect of inoculum formulation and pollutant bioavailability in solid matrices. J. Hazard. Mater..

[70] Sarkar P., Roy A., Pal S., Mohapatra B., Kazy S.K., Maiti M.K., Sar P. (2017). Enrichment and characterization of hydrocarbon-degrading bacteria from petroleum refinery waste as potent bioaugmentation agent for in situ bioremediation. Bioresour. Technol..

[71] Sarkar J., Kazy S.K., Gupta A., Dutta A., Mohapatra B., Roy A., Bera P., Mitra A., Sar P. (2016). Biostimulation of Indigenous Microbial Community for Bioremediation of Petroleum Refinery Sludge. Front. Microbiol..

[72] Kostka J.E., Prakash O., Overholt W.A., Green S.J., Freyer G., Canion A., Delgardio J., Norton N., Hazen T.C., Huettel M. (2011). Hydrocarbon-Degrading Bacteria and the Bacterial Community Response in Gulf of Mexico Beach Sands Impacted by the Deepwater Horizon Oil Spill. Appl. Environ. Microbiol..

